# Conditions for Multi-functionality in a Rhythm Generating Network Inspired by Turtle Scratching

**DOI:** 10.1186/s13408-015-0026-5

**Published:** 2015-07-17

**Authors:** Abigail C. Snyder, Jonathan E. Rubin

**Affiliations:** Department of Mathematics, University of Pittsburgh, 301 Thackeray Hall, Pittsburgh, PA 15260 USA

**Keywords:** Central pattern generator, Turtle motor rhythms, Phase plane analysis, Slow dynamics

## Abstract

Rhythmic behaviors such as breathing, walking, and scratching are vital to many species. Such behaviors can emerge from groups of neurons, called central pattern generators, in the absence of rhythmic inputs. In vertebrates, the identification of the cells that constitute the central pattern generator for particular rhythmic behaviors is difficult, and often, its existence has only been inferred. For example, under experimental conditions, intact turtles generate several rhythmic scratch motor patterns corresponding to non-rhythmic stimulation of different body regions. These patterns feature alternating phases of motoneuron activation that occur repeatedly, with different patterns distinguished by the relative timing and duration of activity of hip extensor, hip flexor, and knee extensor motoneurons. While the central pattern generator network responsible for these outputs has not been located, there is hope to use motoneuron recordings to deduce its properties. To this end, this work presents a model of a previously proposed central pattern generator network and analyzes its capability to produce two distinct scratch rhythms from a single neuron pool, selected by different combinations of tonic drive parameters but with fixed strengths of connections within the network. We show through simulation that the proposed network can achieve the desired multi-functionality, even though it relies on hip unit generators to recruit appropriately timed knee extensor motoneuron activity, including a delay relative to hip activation in rostral scratch. Furthermore, we develop a phase space representation, focusing on the inputs to and the intrinsic slow variable of the knee extensor motoneuron, which we use to derive sufficient conditions for the network to realize each rhythm and which illustrates the role of a saddle-node bifurcation in achieving the knee extensor delay. This framework is harnessed to consider bistability and to make predictions about the responses of the scratch rhythms to input changes for future experimental testing.

## Introduction

**Fig. 1:**
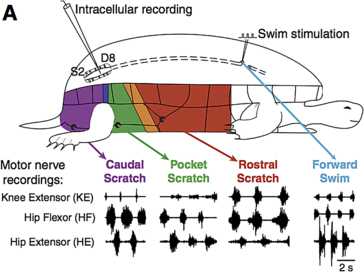
Schematic illustration of stimulation of different turtle body sites. Illustration of how stimulation of different sites, via an electrode for swim or body surface contact for scratch, elicits different patterns of activity in motoneuron recordings from turtle. Figure source: [1]

Under experimental conditions, intact turtles are observed to generate a variety of rhythmic motor patterns corresponding to stimulation of different body regions (including caudal scratch, rostral scratch, pocket scratch, and forward swim; see Fig. 1) [1]. All of these patterns feature alternating phases of motoneuron activation that occur repeatedly, while different patterns are distinguished by the relative timing and duration of activity of hip extensor motoneurons, hip flexor motoneurons and knee extensor motoneurons. Notably, these stable, rhythmic behaviors arise in the absence of rhythmic stimulation, suggesting that a central pattern generator (CPG) may be responsible. Spinalized turtles, in which motor pathways from higher brain areas have been cut, display corresponding fictive behaviors in response to the same forms of stimulation, which suggests that necessary components for rhythm generation are present in the brain stem and spinal cord [1–4]. However, even with restriction to these areas, the complexity of the neuronal networks in turtle have made it impractical to locate the relevant CPG neurons experimentally.

As an alternative, researchers have, on theoretical grounds, proposed structures that may represent important components or principles involved in the function of the relevant CPGs [5–9]. Computational methods offer a natural means to investigate these structures’ properties and generate predictions about them that may guide future experimental investigations. In this work, we use computational methods to study a model CPG network that was previously suggested as a kernel for turtle pocket scratch (pocket) and rostral scratch (rostral) motor pattern generation [4]. Specifically, we demonstrate that a simulated version of this model can generate both of these rhythms, selected only by the relative levels of certain constant inputs, for fixed parameter values, and we derive conditions on model parameters that ensure that this dual functionality will exist.

Previous theoretical work on motor pattern generation in turtles [5, 10] focused on the generation of two other turtle motor rhythms, caudal scratch and forward swim, from a variety of network architectures, testing their compatibility with several observed experimental characteristics. A common theme between those works and this one is the notion of eliciting multiple rhythms from a fixed network. Indeed, both approaches depart from the traditional unit pattern generator framework (in which there exist specific excitatory and inhibitory populations dedicated to controlling the activity of motoneurons associated with each joint, [11]). The models in the earlier paper included distinct interneurons projecting to each motoneuron (MN) involved, but these could interact directly in the rhythm generation process; furthermore, inhibition was restricted to interactions shaping the interneuron outputs, rather than impinging on MNs directly [5]. Here, we do not maintain a complete segregation of projection targets and instead show that by considering only hip-related pools of excitatory and inhibitory interneurons, each projecting to both hip and knee MNs, appropriate knee-hip timing relations can be produced.

This result may seem surprising in light of past theory; however, a variety of experimental works [2–4, 12] have shown that knee extensor MNs receive temporally overlapping excitation and inhibition and that the time courses of the inputs to knee extensor MNs are similar to those of inputs to hip flexor MNs in rostral and to hip extensor MNs in pocket. Berkowitz and Stein argued that an architecture featuring excitatory and inhibitory pools of interneurons for each of hip extensor and hip flexor (with each MN population active in synchrony with its respective excitatory pool), which also project to knee extensor MNs, could be more consistent with experimental findings than other architectures [4]. The idea that different rhythm generators can control knee extensor MN timing in different rhythms also fits in with recent observations from experiments in the mouse hindlimb locomotor network, which suggest that intrinsically rhythmic interneuron modules can be flexibly recruited to drive MN pools [13]. Certainly, knee flexor motoneurons are also involved in the generation of these rhythms [9, 14, 15]. Hip extension, hip flexion, and knee extension are sufficient to typify the rhythms, however, and previous studies have focused on these three MN populations [1–4], so we do not consider hip flexor activity in this work.

While the specific network architecture that we consider is motivated by findings from experiments in turtles, our model has a variety of features that are interesting from a mathematical point of view and that may be of use in other modeling work. Wherever possible, we use a general framework and mathematical approach to gain insight into the mechanisms underlying our key results: a single network can (in a nontrivial way) produce two distinct rhythms selected by constant input levels, the timing of activation of a neuron receiving concurrent excitation and inhibition at all times can be controlled by different inputs under different conditions, and a delay in the onset of activity of one neuron relative to another can arise robustly in a model network lacking any explicit inclusion of delay. Our general mathematical approach will allow our findings, while made in a model for turtle motor rhythm generation, to be extensible to other networks with fairly general features.

The remainder of this paper is organized as follows. In Sect. 2, we present the details of the implemented architecture and the specific mathematical choices made to model it. Section 3 has three main parts. First, we show results of simulations that illustrate the multi-functionality of the model network (Sect. 3.1). Next, we derive a reduced slow phase space based on knee extensor motoneuron dynamics in which analysis becomes tractable and apply this framework to elucidate the fundamental mechanisms that generate the network dynamics we observe (Sect. 3.2). Finally, we harness the phase space to consider additional experimental findings and new predictions relating to bistability and to responses to changes in inputs (Sect. 3.3). The paper concludes with a discussion (Sect. 4).

## Model

**Fig. 2:**
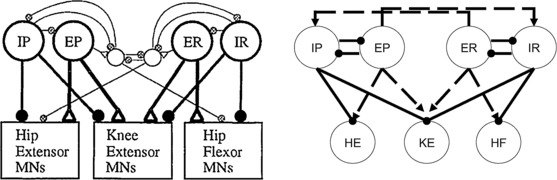
Proposed (*left*) and implemented (*right*) network architectures. *Solid circles* correspond to inhibitory synaptic connections, *open triangles* (*left*) and *dashed arrows* (*right*) to excitatory ones. Figure source for proposed architecture: [4]

A possible motor CPG architecture, differing from the traditional unit pattern generator (UPG) framework with a separate interneuron pool driving each muscle’s motoneurons [11, 16], was proposed based on experimental results on turtle scratching rhythms [4] (Fig. 2, left). As has been well established, however, drawing a plausible wiring diagram for a rhythmic circuit does not allow the immediate inference of actual circuit activity patterns [17]. To explore network dynamics, we implement a simplified version of the proposed architecture, featuring a layer of interneuron pools indexed by labels $i \in\{\mathit {IP}, \mathit {EP}, \mathit {ER}, \mathit {IR}\} $ interacting with each other and feeding forward to a layer of MNs indexed by labels $i \in\{\mathit {HE}, \mathit {KE}, \mathit {HF}\}$ that do not interact. In lieu of an excitatory pool exciting an inhibitory sub-population that in turn inhibits or disinhibits inhibitory pools as originally proposed (e.g. *EP* excites a sub-population that inhibits *IP* and disinhibits *IR*, Fig. 2, left), in our model E and I pools are linked, for simplicity, via direct synaptic connections (Fig. 2, right). A variety of notation associated with this model and its dynamics will be introduced throughout the paper, which we summarize in Table 1.

**Table 1:** **Variables**

$V_{i}$	Membrane potential for population *i*
$h_{i}$	Deinactivation of persistent sodium current for population *i*
$s_{i}$	Slow synaptic gating variable for population *i*
$I_{\mathit {Na}P}$	Persistent sodium current
$I_{\mathrm {syn}}$	Synaptic input from the network
$I_{\mathrm {ext}}$	External synaptic input
$F(V_{i}, h_{i}, s_{i})$	Right hand side of the voltage differential equation
$g_{i}(V_{i},h_{i})$	Right hand side of the persistent sodium differential equation
$g_{\mathrm {syn}}^{i,j}$	Synaptic weight of the synapse from population *j* to population *i*
$i_{i}^{\mathrm {ext}}$	Weight of external drive to population *i*
**s**	Vector of all synaptic variables in the network
$V_{i,X}(h,\mathbf{s})$	Left (*X* = L), middle (*X* = M) or right (*X* = R) branch of the cubic voltage nullcline for population *i*
$p_{i,X}(\mathbf{s})$	Fixed point located on the *X*∈{L,M,R} branch of the voltage nullcline for population *i*
$(V_{i}^{\mathrm {JU}}(\mathbf{s}),h_{i}^{\mathrm {JU}}(\mathbf{s}))$	Jump up curve, curve in slow phase space from which population *i* may enter the active phase
$(V_{i}^{\mathrm {JD}}(\mathbf{s}),h_{i}^{\mathrm {JD}}(\mathbf{s}))$	Jump down curve, curve in slow phase space from which population *i* may enter the silent phase
$s_{\max}$	Maximum value achieved by synaptic gating variable
$s_{\mathrm {dynamic}}$	Synaptic gating variable evolving in time for a given portion of the rhythm, while the other synaptic gating variable is fixed
$I=\{i_{\mathit {IP}}^{\mathrm {ext}}, i_{\mathit {EP}}^{\mathrm {ext}}, i_{\mathit {ER}}^{\mathrm {ext}}, i_{\mathit {IR}}^{\mathrm {ext}} \}$	Set of external drives to populations of interneurons
$T_{\mathrm {active}}^{j}(I)$	Length of time population *j* is active for a given *I*
$s_{\mathrm {SN}}$	Value of *s* at which saddle-node bifurcation occurs
$s_{\mathit {ER}}^{\min}(I)$	Minimum value achieved by $s_{\mathit {ER}}$ for a given *I*
$I_{s}= [ s_{\mathit {ER}}^{\min}(I),s_{\mathrm {SN}} ]$	Values of $s_{\mathit {ER}}$ from which *KE* can enter the active phase
$h_{\max}$	$h_{\mathit {EP}}^{\mathrm {JD}}(s_{\max}) = h_{\mathit {ER}}^{\mathrm {JD}}(s_{\max})$, the largest $h_{\mathit {KE}}$ value at which *KE* can enter the silent phase
$h_{\min}(I)$	$h_{\mathit {ER}}^{\mathrm {JD}}(s_{\mathit {ER}}^{\min}(I))$, the value of $h_{\mathit {KE}}$ on the *ER* curve of jump down knees corresponding to $s_{\mathit {ER}}^{\min}(I)$
$I_{h}= [ h_{\min}(I),h_{\max} ]$	Values of $h_{\mathit {KE}}$ at which *KE* can enter the silent phase
$\mathit {LK}_{I_{s}}$	The part of the curve of jump up knees corresponding to $s \in I_{s}$
*T*(*I*)	Time for *s* to decay from $s_{\mathrm {SN}}$ to $s_{\mathit {ER}}^{\min}(I)$
*h*(*a*;*b*,*c*)	$h_{\mathit {KE}}$ value at time *a* for a trajectory that started at time 0 with initial condition $(h_{\mathit {KE}},s)=(b,c)$
$h_{\mathit {ER}}^{\min}(I)$	*h* value on the *ER* jump up curve given by $s_{\mathit {ER}}^{\min}(I)$
$h_{\mathrm {SN}}^{+}$	Forward flow of $(h_{\mathrm {SN}}, s_{\mathrm {SN}})$ for time *T*(*I*)
$h_{\mathrm {SN}}^{-}$	Backward flow of $(h_{\mathrm {SN}}, s_{\mathrm {SN}})$ to the line $s=s_{\max}$
$h_{s_{\min}}^{-}$	Backward flow of $(h_{\mathit {ER}}^{\mathrm {JU}}(s_{\mathit {ER}}^{\min}(I)), s_{\mathit {ER}}^{\min}(I))$ to the line $s=s_{\max} $
$t^{*}$	Minimal time spent in the silent phase by *KE*

Based on the experimental recordings shown in Fig. 1 and the architecture in Fig. 2, the parsimonious assumptions are that *HE* activates in synchrony with its excitatory interneuron population *EP*, which activates in antiphase with the inhibitory interneuron population *IP*, while *HF* activates in synchrony with its excitatory interneuron population *ER*, which activates in antiphase with the inhibitory interneuron population *IR*. The nature of the rhythms (Fig. 1) indicates additionally that *HE* and *HF* must activate in antiphase for both rhythms, with *HF* activated longer in rostral and *HE* activated longer in pocket. It was hypothesized that *KE* receives inputs that are similar to those received by *HF* in rostral and similar to those received by *HE* in pocket [3]. The subsequently proposed architecture in Fig. 2, however, suggests that the inputs to *KE* are proportional to those to both *HE* and *HF*, which makes it less clear why *KE* synchronizes with *HF*, after some delay, in rostral and with *HE* in pocket (Fig. 1), which is what we seek to explain.

Since we seek to assess the basic rhythm generating capabilities of the proposed architecture, we model each neuronal population in the network as a single cell, leaving issues of heterogeneity for future investigation; we nonetheless refer to each as a “population” in the remainder of the paper (cf. [6]). Inasmuch as the relevant rhythm generating neurons in turtle have not been identified, the specific currents that are central to their rhythmicity are not known. Given this situation, it makes sense to avoid overly specific assumptions about the dynamics of model components. The dynamically simple Wilson–Cowan equations were used in related previous work [5] to model forward swim and caudal scratch rhythms. However, there is a delay in the onset of knee extensor activity relative to hip extensor in caudal scratch that was not modeled in the earlier study. Since the delay of knee extensor onset in rostral scratch is one of the key features that we seek to model, and phase plane considerations suggest that the monotone nullclines of a Wilson–Cowan system cannot give a significant delay, the Wilson–Cowan framework does not appear to be appropriate for our study.

As an alternative, we use a minimal Hodgkin–Huxley type model for each population. We choose an inward, slowly deinactivating persistent sodium current ($I_{\mathit {Na}P}$) as the primary current controlling oscillations in our model. This current has been used in previous CPG modeling studies [6, 7, 18, 19] has been observed experimentally in neurons in other CPGs [20], and is well suited to supply the voltage plateaus underlying bursts of spikes. Since past computational and mathematical work has established that certain classes of currents endow models with similar properties, this specific current choice is not critical for qualitative aspects of our model’s behavior, and our results will apply immediately to networks featuring other inward, slowly deinactivating currents [18, 21]. We omit the details of actual spikes in our model, since the relative durations of active periods, not specific spiking dynamics, are the primary results that we seek to reproduce and since plateau potentials are observed in turtle motoneurons [22, 23]. As a result, we obtain an analytically tractable framework, which would not be possible from incorporation of detailed models for turtle motoneuron dynamics [23, 24].

Given these considerations, our model for each interneuron population takes the form

1 $$ \begin{aligned} C_{m} \dot{V_{i}} &= -I_{\mathit {Na}P}(V_{i},h_{i})-I_{L}(V_{i})- \sum_{j \ne i}I_{\mathrm {syn}}(V_{i},s_{j})-I_{\mathrm {ext}}(V_{i}) \equiv F_{i}(V_{i},h_{i},\mathbf{s}), \\ \dot{h_{i}} &= \bigl(h_{\infty}(V_{i})-h_{i} \bigr)\tau_{h}(V_{i}) \equiv g_{i}(V_{i}, h_{i}), \\ \dot{s_{i}} &= \alpha(1-s_{i})s_{\infty}(V_{i})- \beta s_{i}, \end{aligned} $$

where $V_{i}$ denotes voltage, $h_{i}$ the inactivation of the persistent sodium current $I_{\mathit {Na}P}$, $s_{i}$ the fraction of the maximal synaptic conductance that is induced by the population’s activity, and **s** the vector of *s* variables of all populations in the network (although the evolution of $V_{i}$ does not depend directly on $s_{i}$). In the voltage equation for population *i*, $I_{\mathit {Na}P}(V_{i},h_{i}) = g_{\mathit {Na}P}m_{\infty}h(V_{i}-e_{\mathit {Na}})$, $I_{L}(V_{i}) = g_{L}(V_{i}-e_{L})$ is a leak current, $I_{\mathrm {syn}}(V_{i},s_{j}) = g_{\mathrm {syn}}^{ij}s_{j}(V_{i}-e_{\mathrm {syn}})$ for $e_{\mathrm {syn}} \in\{e_{\mathrm {syn}}^{\mathrm {exc}}, e_{\mathrm {syn}}^{\mathrm {inh}} \}$ denotes synaptic current induced by population *j*, $I_{\mathrm {ext}}(V_{i}) = (i^{\mathrm {ext}}_{i})(V_{i}-e_{\mathrm {syn}}^{\mathrm {exc}})$ denotes excitatory synaptic current with conductance $i^{\mathrm {ext}}_{i}$ from a source outside the network, $m_{\infty}$, $h_{\infty}$, and $s_{\infty}$ are monotone sigmoidal functions given by $x_{\infty}(v) = (1+\exp((v-x_{\mathrm {half}})/\theta _{x}))^{-1}$, $x\in\{m,h,s\}$ with $m_{\infty}$ and $s_{\infty}$ increasing and $h_{\infty}$ decreasing, and $\tau_{h}(v) = \epsilon \cosh((v-h_{\mathrm {half}})/2\theta_{h})$ for $0 < \epsilon\ll1$. All synaptic inputs are defined with $g_{\mathrm {syn}}^{ij}>0$; whether a synaptic input is excitatory or inhibitory is determined by its reversal potential $e_{\mathrm {syn}}$. Default parameter values used in simulations are listed in Table 2; values of $i^{\mathrm {ext}}_{i}$ are varied and are discussed as they arise in our analysis. Simulations of the above system give physiologically realistic voltage ranges with the parameters used in Table 2. However, because we are interested in relative durations of activity, it is more useful to consider rescaled voltage as a representation of population activity. That is, the population activity, PA, is related to voltage, *V*, as follows: $\operatorname {PA}(V) = 1 / (1+e^{ (V+30)/{-2} } ) $. This can be seen in Figs. 6, 15, and 16.

**Table 2:** **Model parameters**

Parameter	Units
$C_{m}$	0.21 *pF*
$g_{\mathit {Na}P}$	10 nS
$e_{\mathit {Na}}$	50 mV
$g_{L}$	2.8 nS
$e_{L}$	−65 mV
$m_{\mathrm {half}}$	−37 mV
$\theta_{m}$	−6 mV
$h_{\mathrm {half}}$	−30 mV
$\theta_{h}$	6 mV
*ϵ*	0.01 ms^−1^
$s_{\mathrm {half}}$	−43 mV
$\theta_{s}$	−0.1 mV
$e_{\mathrm {syn}}^{\mathrm {inh}}$	−80 mV
$e_{\mathrm {syn}}^{\mathrm {exc}}$	0 mV
*α*	1
*β*	0.08

With these parameter values, our model equations satisfy several structural hypotheses. We base our analytical arguments on these hypotheses, so that our results extend beyond our specific choices of model functions and parameter values.

1. For each population *i*, for all relevant synaptic inputs **s**, the $V_{i}$ nullcline, $\{ (V_{i}, h_{i}) : F_{i}(V_{i},h_{i},\mathbf{s})=0\}$, is cubic in the $(V_{i},h_{i})$ phase plane. This nullcline includes left, middle, and right branches, denoted, respectively, by $V=V_{i,\mathrm{L}}(h,\mathbf{s})$, $V=V_{i,\mathrm{M}}(h,\mathbf{s})$, and $V=V_{i,\mathrm{R}}(h,\mathbf{s})$ with $V_{i,\mathrm{L}} < V_{i,\mathrm{M}}< V_{i,\mathrm{R}}$ for each $(h,\mathbf{s})$ for which all three exist. For our choice of model, for fixed **s**, $V_{i,\mathrm{L}}$ and $V_{i,\mathrm{R}}$ increase as a function of *h* and $V_{i,\mathrm{M}}$ decreases as a function of *h*, so this will henceforth be assumed as well, although it is not required for our results to hold. Figure 3 illustrates these structures and those introduced in subsequent hypotheses.

**Fig. 3:**
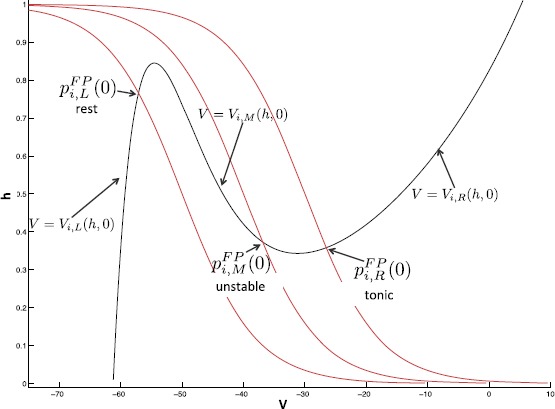
Nullcline configurations for varying values of $\theta_{h}$ (shifting the *h* nullcline, *red*) to illustrate key structures in phase space
2. For each population *i*, the $h_{i}$ nullcline, $\{ (V_{i},h_{i}) : g_{i}(V_{i}, h_{i}) =0 \}$, is monotone decreasing.
3. In the absence of synaptic coupling ($g_{\mathrm {syn}}=0$), each population has a unique fixed point, $p_{i,\mathrm{R}}^{\mathrm {FP}}(0)=(V_{i,\mathrm{R}}^{\mathrm {FP}}(0),h_{i,\mathrm{R}}^{\mathrm {FP}}(0))$, on the right branch of the $V_{i}$ nullcline for a range of input conductances, $i^{\mathrm {ext}}_{i}$.
4. In the presence of coupling ($g_{\mathrm {syn}}>0$) and with input strength $i^{\mathrm {ext}}_{i}$ fixed within the range we consider, the right fixed point is retained and left $p_{i,\mathrm{L}}^{\mathrm {FP}}( \mathbf{s})=(V_{i,\mathrm{L}}^{\mathrm {FP}}(\mathbf{s}),h_{i,\mathrm{L}}^{\mathrm {FP}}(\mathbf{s}))$ and middle $p_{i,\mathrm{M}}^{\mathrm {FP}}(\mathbf{s})=(V_{i,\mathrm{M}}^{\mathrm {FP}}(\mathbf{s}),h_{i,\mathrm{M}}^{\mathrm {FP}}( \mathbf{s}))$ fixed points are gained and lost via saddle-node bifurcations that occur for some nonzero choices of the synaptic input **s** (for example, see Fig. 4).

**Fig. 4:**
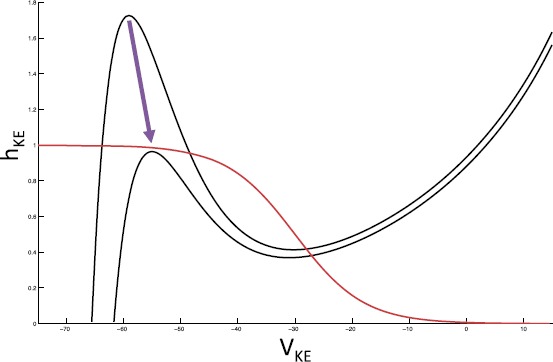
Saddle-node bifurcation for *KE*. *The red curve* is the $h_{\mathit {KE}}$ nullcline, while *the black curves* are $V_{\mathit {KE}}$ nullclines for differing combinations of synaptic input. The change between these two combinations induces a saddle-node bifurcation. We illustrate this bifurcation in the $(V_{\mathit {KE}},h_{\mathit {KE}})$ phase plane since it is critical for delaying *KE* activation in the rostral rhythm

These hypotheses restrict the system such that it has between one and three fixed points for all relevant inputs and coupling strengths. Fixed points on the right branch of the $V_{i}$ nullcline correspond to tonic spiking behavior (since the model lacks spike generating currents), while fixed points on the left branch of the $V_{i}$ nullcline correspond to a relatively constant low voltage. Therefore, hypothesis (H3) means each population is intrinsically tonically active (Fig. 3, right fixed point). In our desired network activity, bursting behavior in a population of neurons consists of regular alternations between states of low voltage near some family of left nullcline branches $V_{i,\mathrm{L}}^{\mathrm {FP}}(\mathbf{s})$ (silent phase) and states of tonic spiking (i.e., elevated voltage) near some family of right nullcline branches $V_{i,\mathrm{R}}^{\mathrm {FP}}(\mathbf{s})$ (active phase), linked via abrupt voltage transitions of significant amplitude, corresponding to jumps between branches. In this framework, the synaptic decay must be sufficiently slow relative to the time scale of voltage jumps, to avoid convergence to a fixed point. Since the synaptic variables represent conductances induced by populations of neurons that are generating a burst of activity, the assumption that they decay gradually during a phase is quite reasonable. On the other hand, we take synaptic activation to occur on the fast time scale, reflecting the synchronized onset of activity in a presynaptic population; see Eqs. (2) and (3) below.

A key point is that hypotheses (H3) and (H4) together imply that transitions from the silent to the active phase must occur by escape. Given a mutually inhibitory pair of populations where one is active and the other is silent, the silent population may become active by reaching the jump up (left) knee of its *V* nullcline (i.e., left fold of its family of *V* nullclines, parameterized by the synaptic strength *s* controlled by the other population). Doing so allows it to jump to the active phase, inhibiting the other population and, for sufficiently large $g_{\mathrm {syn}}$, relegating the other population to the silent phase. When these conditions are met, the two populations form a half-center oscillator in which switches between phases are controlled by the silent population [25, 26]. Thus, in addition to the surfaces of fixed points for each population, $p_{i,X}^{\mathrm {FP}}(\mathbf{s})=(V_{i,X}^{\mathrm {FP}}(\mathbf{s}), h_{i,X}^{\mathrm {FP}}( \mathbf{s}))$, $X \in\{\mathrm{L},\mathrm{M},\mathrm{R}\}$, of mathematical importance are also the surfaces of jump up and jump down *V* nullcline folds, or knees, for each population: $(V_{i}^{\mathrm {JU}}(\mathbf{s}),h_{i}^{\mathrm {JU}}(\mathbf{s}))$ and $(V_{i}^{\mathrm {JD}}(\mathbf{s}),h_{i}^{\mathrm {JD}}(\mathbf{s}))$. For fixed levels of external and synaptic inputs, the jump up (down) knee corresponds to a local maximum (minimum) of the $V_{i}$ nullcline. A surface of knees is then the surface of these local extrema, parameterized by the values of the synaptic input variables, for a fixed external input strength.

Based on our parameter choices (Table 2), for each *i*, we consider that jumps between branches of a *V* nullcline occur instantaneously relative to the rate of $I_{\mathit {Na}P}$ (de)inactivation and relative to the slow decay of $s_{i}$ (set by the small value of *β*) in the silent phase. Furthermore, we have performed simulations with a very steep synaptic activation function $s_{\infty}(v)$, since $\theta_{s}$ is quite small. Thus, for purposes of analysis, we write $\beta= \epsilon\tilde {\beta}$, define $\tau=\epsilon t$, and let a prime denote differentiation with respect to *τ*. We then extract from system (1) in the $\epsilon\to0$ limit a fast subsystem governing jumps between phases:

2 $$ \begin{aligned} C_{m}\dot{V_{i}} &= F_{i}(V_{i},h_{i},\mathbf{s}),\quad j \ne i, \\ \dot{h_{i}} &= 0, \\ \dot{s_{i}} &= \alpha(1-s_{i})s_{\infty}(V_{i}), \end{aligned} $$

a slow subsystem governing evolution within the silent phase:

3 $$ \begin{aligned} h_{i}' &= g_{i}\bigl(V_{i,\mathrm{L}}(h_{i},\mathbf{s}), h_{i} \bigr), \\ s_{i}' &= -\tilde{\beta} s_{i}, \end{aligned} $$

and a slow subsystem governing evolution within the active phase

4 $$ \begin{aligned} h_{i}' &= g_{i}\bigl(V_{i,\mathrm{R}}(h_{i},\mathbf{s}), h_{i} \bigr), \\ s_{i} & = 1. \end{aligned} $$

At any time when there is no population making a fast jump, the collection of populations evolves in a high-dimensional slow phase space with governing equations given by making an appropriate choice of either Eq. (3) or Eq. (4) for each population.

Suppose we consider a collection of *N* interacting populations. Since $s_{i}$ does not affect $V_{i}$, $h_{i}$ directly, it is useful to project the trajectory to an *N*-dimensional slow phase space for each population, with dimensions corresponding to that population’s *h* variable along with the *s* variables for the other $N-1$ populations. The population’s jump up and jump down knees, $(V_{i}^{\mathrm {JU}}( \mathbf{s}),h_{i}^{\mathrm {JU}}(\mathbf{s}))$ and $(V_{i}^{\mathrm {JD}}(\mathbf{s}),h_{i}^{\mathrm {JD}}(\mathbf{s}))$, are then given by surfaces in its slow phase space (e.g. [27, 28]).

In the singular limit, each $s_{i}$ jumps to 1 at the instant (with respect to the slow time scale) of the jump in $V_{i}$, hence the equation $s_{i}=1$ in (4). In our simulations, we will be away from the singular limit and hence the maximal value of *s* is $\alpha/(\alpha+\beta)$, which we will denote by $s_{\max}$ in the analysis below.

## Results

### Baseline Simulation Results

We simulated system (1) using XPPAUT [29] to find parameter values for which the network (Fig. 2, right) would generate a rostral scratch rhythm under one set of constant external input strengths, $\{ i^{\mathrm {ext}}_{i} \}_{\mathcal{R}}$, and a pocket scratch rhythm under a different set of constant external input strengths, $\{ i^{\mathrm {ext}}_{i} \}_{\mathcal{P}}$ (see Fig. 1). We required that synaptic weights, $\{ g_{\mathrm {syn}}^{i j}\}$, were fixed at the same values for both rhythms, such that our results would represent activation of a fixed network by two different forms of stimulation, presumably representing effects of body surface stimulation in two different regions (Fig. 1).

**Fig. 5:**
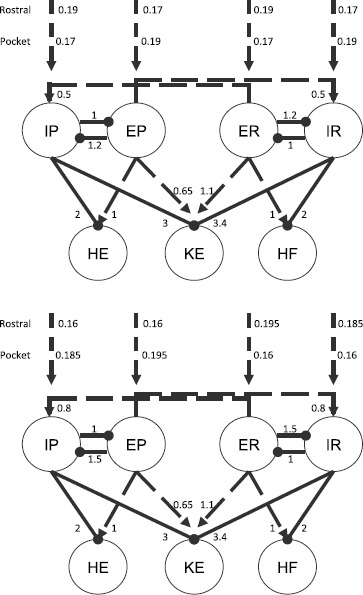
Synaptic weights and input strengths. Two different sets of synaptic weights $g_{\mathrm {syn}}^{i j}$ and external input strengths $i^{\mathrm {ext}}_{i}$ used in our simulations of system (1), with units (mS) omitted. *Top*: “standard” weights; *bottom*: “strong cross-excitation” weights. *Solid lines ending in circles* denote inhibitory connections; *dashed lines ending in arrows* represent excitatory ones. Both sets of weights include certain symmetries but the activity they support is robust to asymmetric perturbations

Two distinct classes of synaptic weights were implemented in the network, standard (S) and strong cross-excitation (SCE) (Fig. 5). The S class is based on the idea that a rostral-inducing stimulus should strongly recruit the excitatory *ER* pool responsible for driving *HF* and less strongly recruit the inhibitory *IR* pool that blocks this action, and similarly for pocket. These input levels can also be interpreted as all four interneuron populations receiving a baseline level of input, with *ER*, *IP* receiving additional input in rostral and *EP*, *IR* receiving additional input in pocket.

The SCE class is based on the reasoning that the entire rostral pool, including both *ER* and *IR*, should be most strongly stimulated by rostral-inducing stimuli, and similarly for pocket. We call this weight class SCE because a stronger cross-excitation from *ER* to *IP* and from *EP* to *IR* (0.8 nS versus 0.5 nS) was used to promote synchrony between these pairs of populations in this case. Here, all four interneuron populations can be viewed as receiving a baseline level of input, but with an additional input boost to the “active side”.

In both cases, the synaptic weights at the interneuron level (not to the MNs) are just a minimal combination that allows oscillations to occur; that is, decreasing any of the weights appreciably without changing the others to compensate leads to a loss of all oscillations. The baseline input strengths (0.17 nS in S and 0.16 nS in SCE) were chosen such that no oscillations are elicited when no interneuron populations receive an additional drive. The S and SCE weights are similar in the sense that they result in qualitatively similar interneuron dynamics and output from the interneurons to the MNs. This output is largely constrained by the required behavior of *HF* and *HE*:

- *HF* and *HE* activate in antiphase and do not receive temporally overlapping excitation and inhibition [2–4] meaning that *IP* must be in antiphase with *EP* and *IR* in antiphase with *ER* (Fig. 2, right panel).
- In light of these antiphase relations, it is natural for *EP*, *IR* to activate in synchrony and *ER*, *IP* to activate in synchrony.
- *HF* is activated longer than *HE* in rostral (Fig. 1, right panel of Fig. 2, Fig. 5), hence *ER* must receive more input than *EP* in rostral (reversed in pocket).

Any synaptic weights selected must satisfy these constraints. Furthermore, as will be seen in the next section, a certain general relationship among the synaptic weights to *KE* must be satisfied to allow both rhythms to be elicited from the network.

**Fig. 6:**
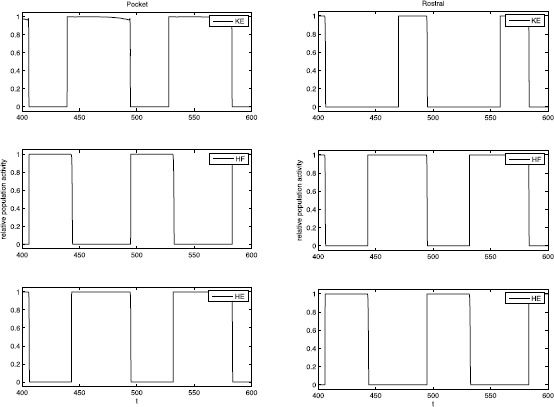
Basic simulation results. Example relative population activity for MN populations resulting from simulation of system (1) with the S weights. MN population identified in *the legend*. *The y-axis* represents population activity as rescaled voltage, *0* indicates silent, *1* indicates active. Note that the relative timing and durations of activity in the simulation match the recordings (see Fig. 1). The SCE weights produce the desired relative timing and durations as well (not shown)

With the S and SCE weights, the network can generate both rostral and pocket rhythms, selected by the external input strengths $\{ i^{\mathrm {ext}}_{i} \}$ as shown in Fig. 5; see Fig. 6 for an example simulation with the S class. Thus, we have confirmed the conjecture that the architecture illustrated in Fig. 2 is capable of such multi-functionality, suggesting its viability as a building block of circuits generating multiple output rhythms from a single set of MNs and muscles. Naturally, for both the S and the SCE weights, there is a range of each input parameter $\{ i^{\mathrm {ext}}_{i} \}$ over which each rhythm persists. As mentioned previously, the reason that both architectures work is because they produce qualitatively similar interneuron activity patterns and corresponding outputs from the interneurons to the MNs; note that the connections from the interneurons to the MNs are weighted the same across both weight classes. The mathematical analysis done in the next section shows that sufficient changes in these interneuron-to-MN weights would cause the network to lose the desired behavior.

### Necessary Conditions for Rhythms

Because hip extensor and hip flexor each only receive antiphase excitation and inhibition and maintain the same antiphase relationship with each other across both rhythms, choosing synaptic weights from the interneuron populations to *HE* and *HF* is easy. We henceforth assume that these weights and the weights within the interneuron network are fixed such that this antiphase behavior, with appropriate relative phase durations, occurs. Because *KE* receives temporally overlapping excitation and inhibition, synchronizes with a different hip component in each rhythm, and exhibits a delay in onset relative to its hip partner in rostral and not pocket, the synaptic weights to *KE* are much more constrained. We will consider dynamics in certain slow phase spaces to derive conditions on these weights that yield multi-functionality of the networks shown in Fig. 5, which generalize to any model with a qualitatively similar structure.

#### Reduction of Slow Phase Space Dimension

To focus on *KE*, we need consider only a subset of the slow variables in the model. *KE* receives four synaptic inputs with conductance variables $\{ s_{\mathit {EP}}, s_{\mathit {ER}}, s_{\mathit {IP}}, s_{\mathit {IR}} \}$, which activate on the fast time scale (Eq. (2)) and decay on the slow time scale (Eq. (3)). Additionally, the inactivation of persistent sodium for *KE*, $h_{\mathit {KE}}$, evolves on the same slow time scale. Therefore, there is a five-dimensional slow phase space for *KE*. Analyzing dynamics in this full, five-dimensional space is impractical.

**Fig. 7:**
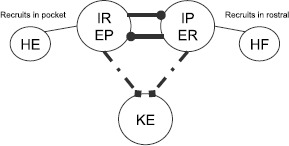
Reduced module controlling knee extensor activity. Two interneuron units form a half-center oscillator, linked by mutual inhibition (*thick solid lines*). Each unit recruits a corresponding hip MN (*thin solid lines*) and supplies a hybrid excitatory and inhibitory input to *KE* (*dot dashed lines with squares*), with a single corresponding synaptic conductance variable

To reduce dimension further, we identify the interneuron pairs that activate together, $(\mathit {EP},\mathit {IR})$ and $(\mathit {ER},\mathit {IP})$, to form a single half-center oscillator and we consider a reduced model to describe *KE* activity, illustrated in Fig. 7. With this reduction, using $e_{\mathrm {syn}}^{\mathrm {exc}}=0$, $s_{\mathit {ER}}=s_{\mathit {IP}}$, and $s_{\mathit {EP}}=s_{\mathit {IR}}$, the synaptic input for knee extensor becomes

$$I_{\mathrm {syn}}^{\mathit {KE}} = s_{\mathit {ER}}\bigl[ (g_{\mathit {IP}}+g_{\mathit {ER}})V_{\mathit {KE}}-g_{\mathit {IP}}e_{\mathrm {syn}}^{\mathrm {inh}} \bigr]+s_{\mathit {EP}}\bigl[ (g_{\mathit {IR}}+g_{\mathit {EP}})V_{\mathit {KE}}-g_{\mathit {IR}}e_{\mathrm {syn}}^{\mathrm {inh}} \bigr]. $$

**Fig. 8:**
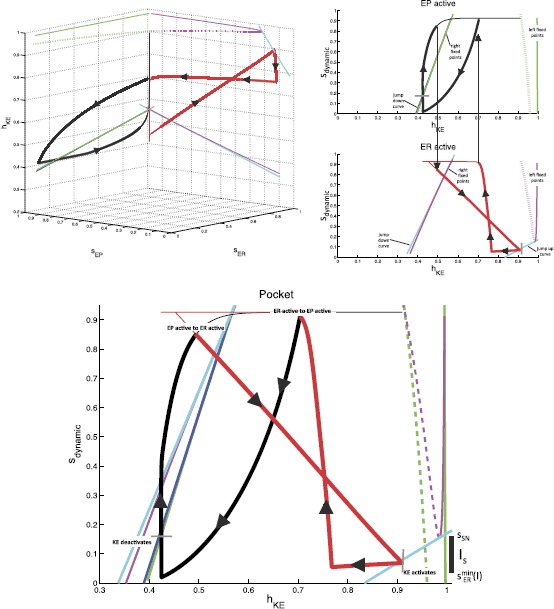
Phase space views for the *KE* dynamics in the reduced module shown in Fig. 7 during the pocket rhythm. *Top left*: full three-dimensional slow phase space. *Top right*: projections onto the two two-dimensional planes where the trajectory lies. *Bottom*: single, combined two-dimensional representation. In all plots, *black and red curves* are projections of parts or all of the trajectory of a periodic pocket scratch solution, with *bold black and thin red* denoting times when *EP* is active and *bold red and thin black* times when *ER* is active. *Green curves* denote the fixed point curves for *KE* $p_{\mathit {KE},\mathrm{R}}^{\mathrm {FP}}(\mathbf{s})$ (stable, *solid*), $p_{\mathit {KE},\mathrm{M}}^{\mathrm {FP}}(\mathbf{s})$ (unstable, *dashed*), and $p_{\mathit {KE},\mathrm{L}}^{\mathrm {FP}}(\mathbf{s})$ (stable, *solid*) (in order of increasing $h_{\mathit {KE}}$) while *EP* is active. *Magenta curves* denote the analogous curves of fixed points for *KE* while *ER* is active. *The dark blue curve* is the curve of jump down knees for *KE* while *EP* is active; *cyan curves* are jump down knees and jump up knees (larger $h_{\mathit {KE}}$ values) for *KE* while *ER* is active. Finally, *dashed black curves in the top right* indicate points on the two projections that correspond to the same times, when the switches between the *EP* active phase and the *ER* active phase occur. *Additional labeling on the top right* indicates relevant structures defined above. *Additional labeling on the bottom* indicates key changes in activity of various populations throughout the rhythms. *Gray tick marks* indicate transitions from activity to silence. This labeling holds for all panels and future figures

This step reduces our phase space from five dimensions to three, with variables $(h_{\mathit {KE}}, s_{\mathit {EP}}, s_{\mathit {ER}})$. The projection of the periodic pocket trajectory of the reduced model to $(h_{\mathit {KE}},s_{\mathit {EP}},s_{\mathit {ER}})$ space is shown in the top left of Fig. 8, along with several curves that are important for understanding *KE* dynamics. These plots are critical to our analysis. When *ER* is active, $s_{\mathit {ER}} \approx s_{\max}$, so the corresponding part of the trajectory, color coded red, lies approximately on the $\{ s_{\mathit {ER}} = s_{\max} \}$ plane within phase space, which is the back right face of the cube shown. Similarly, the epoch with *EP* active has $s_{\mathit {EP}} \approx s_{\max}$ and yields a trajectory, color coded black, near the back left face of the cube. As an alternative to considering a three-dimensional phase space, however, it is convenient to switch between a pair of two-dimensional slow phase planes, corresponding to the back two faces in the top left of Fig. 8, as *EP* and *ER* alternate between periods of silence and activity. These are shown in the top right of Fig. 8. For example, while *EP* is active, $s_{\mathit {ER}}$ evolves and the projection of the trajectory to the $(h_{\mathit {KE}},s_{\mathit {ER}})$ plane is shown as the thick black curve. Of course, even after *EP* switches from active to silent, the projection of the trajectory to the $(h_{\mathit {KE}},s_{\mathit {ER}})$ plane still exists; the projected trajectory segment after the switch is shown as the thin black curve. Using similar considerations for the projection to $(h_{\mathit {KE}},s_{\mathit {EP}})$, we in fact plot two copies of the full trajectory, each in its own two-dimensional phase plane, one with the trajectory shown thick while *EP* is active and thin while *ER* is active, and the other the opposite. The switch from *EP* active to *ER* active occurs abruptly when $s_{\mathit {EP}}$ begins its slow decay from $s_{\max}$ and $s_{\mathit {ER}}$ increases very rapidly (instantly in the singular limit) to $s_{\max}$, and we switch each curve from thick to thin when $s_{\mathit {EP}}=s_{\mathit {ER}}$ occurs.

Finally, since the values over which $s_{\mathit {ER}}$ and $s_{\mathit {EP}}$ vary over each period are similar, both slow phase planes can be compressed to a single plot. Again, when this plot is displayed in the bottom part of Fig. 8, we show two copies of the trajectory. For the black (red) copy, $s_{\mathrm {dynamic}}$ should be interpreted as $s_{\mathit {ER}}$ ($s_{\mathit {EP}}$), with thick and thin parts as in the separate two-dimensional plots (thick black when *EP* is active such that $s_{\mathit {ER}}$ decays gradually, thick red when *ER* is active such that $s_{\mathit {EP}}$ decays gradually).

For fixed input levels $(s_{\mathit {EP}},s_{\mathit {ER}})$, the $V_{\mathit {KE}}$ nullcline has one or more fixed points, a jump up knee, and a jump down knee. These become two-dimensional surfaces under variation of both inputs, while fixing one input at $s_{\max}$ selects a one-dimensional curve. In Fig. 8, the curves of fixed points for $s_{\mathit {EP}}=s_{\max}$ are shown in green and for $s_{\mathit {ER}}=s_{\max}$ in magenta; both show up in the bottom plot, but it is important to keep in mind that each is only meaningful when $s_{\mathrm {dynamic}}$ has the correct interpretation. Similarly, the curves of knees are shown in dark blue and cyan. There are two cyan curves, with smaller $h_{\mathit {KE}}$ values for jump down knees than for jump up. There is only one dark blue curve because the curve of jump up knees is outside of the relevant range of $(h_{\mathit {KE}},s)$ values when *EP* is active.

#### Scratch Trajectories and Weights of Synapses onto *KE*

To generate pocket and rostral scratch rhythms in our model, we had to select values for synaptic connections in the model network, which remain the same for both rhythms, and strengths of external inputs to the network, which differ between the rhythms. As mentioned previously, fixing the weights of synapses to the *HE* and *HF* MNs is not particularly interesting, since the desired antiphase activation patterns for each rhythm are set at the interneuron level in the full or reduced model. For convenience, we simply choose $g_{\mathrm {syn}}^{\mathit {HE}, \mathit {EP}}=g_{\mathrm {syn}}^{\mathit {HF}, \mathit {ER}}$ and $g_{\mathrm {syn}}^{\mathit {HE}, \mathit {IP}}=g_{\mathrm {syn}}^{\mathit {HF}, \mathit {IR}}$.

**Fig. 9:**
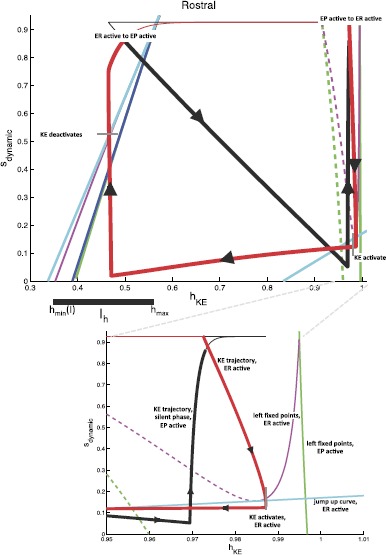
Rostral slow phase plane. Trajectory for *KE* for rostral scratch projected to a single slow phase plane. *Coloring of curves* is identical to Fig. 8. *Bottom*: zoomed view near the saddle-node bifurcation where the fold in *the magenta* fixed point curve intersects *the cyan* jump up knee curve for $\mathit {ER}/\mathit {HF}$ active

The weights of synapses onto *KE* are more interesting. To understand how these are constrained, we can focus on the reduced model, which maintains four distinct synaptic weights from the interneurons onto *KE*. With the convenient viewpoint that we have established, it is now helpful to consider the details of the trajectories for pocket scratch (Fig. 8) and rostral scratch (shown in Fig. 9 in a two-dimensional view analogous to the bottom panel of Fig. 8) for our baseline parameter choices. Recall that in the pocket rhythm, *KE* activates with *HE*, here represented by the activation of *EP*. When *EP* becomes active and the thick black part of the trajectory starts, $h_{\mathit {KE}}$ decreases, corresponding to the trajectory being in the active phase for *KE*, near a right branch of the $V_{\mathit {KE}}$ nullcline. The trajectory cannot cross the curve of jump down knees (dark blue) with $s_{\mathrm {dynamic}}$ decreasing, because it is blocked by the green fixed point curve (which almost coincides with the dark blue one in Figs. 8 and 9). The switch of $s_{\mathrm {dynamic}}$ from decreasing to increasing corresponds to the activation of *ER* (and hence *HF*). The rise in $s_{\mathrm {dynamic}}$ pulls the trajectory across the curve of jump down knees of the $V_{\mathit {KE}}$ nullcline (dark blue), terminating the active phase of *KE*. We then switch our view to the thick red trajectory, along which $h_{\mathit {KE}}$ increases (and $s_{\mathrm {dynamic}}=s_{\mathit {EP}}$ decreases), corresponding to the trajectory being in the silent phase for *KE*, near a left branch of the $V_{\mathit {KE}}$ nullcline. The trajectory actually reaches the curve of jump up knees (cyan), and hence *KE* activates before the activation of *EP* and *HE* cause $s_{\mathrm {dynamic}}=s_{\mathit {EP}}$ to increase. But shortly after this switch, *EP* itself activates, yielding a rise in $s_{\mathrm {dynamic}}$, and we switch back to the thick black trajectory, where we started. In fact, experiments reveal a natural variability in pocket scratch patterns. There are many experimental examples of pocket rhythms in which knee extensor becomes active just before hip extensor, at the final moments of hip flexor activity, and indeed a mean pocket rhythm computed from experimentation has this property [30]. Hence, this result provides validation that the solution that we have obtained provides a reasonable reduced representation of a pocket rhythm.

In the rostral rhythm, *KE* activation follows that of *HF*, here represented by the activation of *ER*, with a delay. When *ER* becomes active, and the thick red part of the trajectory starts, *KE* is still in the silent phase, with a fixed point on the left branch of the $V_{\mathit {KE}}$ nullcline (solid magenta line at the far right of Fig. 9; see especially the bottom panel of Fig. 9). As $s_{\mathrm {dynamic}}$ decreases, the trajectory approaches the corresponding branch of fixed points, and *KE* cannot activate until this branch undergoes a saddle-node bifurcation (meeting the dashed fixed point branch in the figure) at the curve of jump up knees of the $V_{\mathit {KE}}$ nullcline (lower right cyan curve; also see Fig. 4). At the bifurcation, *KE* activates and $h_{\mathit {KE}}$ starts to decay, with the trajectory heading toward the magenta curve of fixed points in the left part of Fig. 9. When the activity of *ER* terminates, $s_{\mathrm {dynamic}}$ increases, which pulls the trajectory through the curve of jump down knees (cyan) and hence switches *KE* to the silent phase. With *EP* now activated (thick black part of the trajectory) and *KE* silent, $h_{\mathit {KE}}$ increases, but there is no curve of jump up knees available to reach over the relevant range of $(h_{\mathit {KE}},s_{\mathrm {dynamic}})$ (note the absence of a dark blue curve in the lower right of Fig. 9, analogous to its absence in Fig. 8). Thus *KE* remains silent until the active phase of *EP* ends, $s_{\mathrm {dynamic}}$ rises, and *ER* activates at the transition from the thick black to the thick red part of the trajectory, where we started.

From our investigations, it appears that obtaining both pocket and rostral scratch rhythms with the same set of synaptic weights through the dynamic mechanisms we have described requires certain phase plane features and timing relations, which arise in the trajectory descriptions we have provided. Classifying these in terms of particular phases of rhythms, the requirements on the trajectory projected to *KE* space are as follows:

(i) pocket, *EP* active: the trajectory must not reach the curve of jump down knees as $s_{\mathrm {dynamic}}$ decreases yet must cross it as $s_{\mathrm {dynamic}}$ rises (Fig. 8, the red part of the trajectory does not increase through the cyan curve but the black part of the trajectory increases through the blue curve);
(ii) pocket, *ER* active: the trajectory must reach the curve of jump up knees as $s_{\mathrm {dynamic}}$ decreases, but only sufficiently late in the active phase of *ER* (Fig. 8, the red part of the trajectory reaches the right cyan curve near where it switches to black);
(iii) rostral, *ER* active: the trajectory must follow a curve of fixed points to a saddle-node bifurcation at the curve of jump up knees, must subsequently not reach the curve of jump down knees as $s_{\mathrm {dynamic}}$ decreases, and must cross the jump down knees as $s_{\mathrm {dynamic}}$ rises (Fig. 9, red parts of the trajectory);
(iv) rostral, *EP* active: the trajectory must not reach the curve of jump up knees as $s_{\mathrm {dynamic}}$ decreases (Fig. 9, note that there is no curve of jump up knees visible while *EP* is active, corresponding to the black part of the trajectory).

The first part of requirement (iii) is critical for imposing a delay between *ER* activation and *KE* activation. Requirement (iv) goes together with (iii); certainly no delay would be possible if the trajectory reached a curve for the activation of *KE* even before *ER* activated at all! To achieve requirements (iii) and (iv), we find that it is necessary but not sufficient for $g_{\mathrm {syn}}^{\mathit {KE}, \mathit {EP}}$, $g_{\mathrm {syn}}^{\mathit {KE}, \mathit {IP}}$, $g_{\mathrm {syn}}^{\mathit {KE}, \mathit {ER}}$, $g_{\mathrm {syn}}^{\mathit {KE}, \mathit {IR}}$ to be such that the $\mathit {ER}/\mathit {IP}$ active pair has an overall more excitatory effect on *KE* than the $\mathit {EP}/\mathit {IR}$ active pair. This means that the synaptic weights coming from *ER* and *IR* to *KE* must be stronger than those coming from *EP* and *IP*. Once these requirements are imposed, we find that *KE* also activates while *ER* is still active in the pocket rhythm; requirement (ii) constrains weights so that this happens as late as possible, providing a realistic pocket rhythm. This is not, however, contrary to many experimental observations. For example, Earhart et al. [30] appear to find this slight overlap. Finally, both requirements (i) and (iii) are partially trivial, since the trajectory is blocked from reaching jump down knees by the location of fixed point curves. Nonetheless, they do constrain weights to ensure that $h_{\mathit {KE}}$ decays sufficiently during each active phase such that subsequent rises in $s_{\mathrm {dynamic}}$ can pull the trajectory across the curves of jump down knees, transitioning *KE* to the silent phase along with its interneuron partner, as desired.

#### Conditions for Rhythm Selection and Slow Phase Plane Analysis/Contraction Arguments

With our synaptic weights onto *KE* and slow phase plane structure fixed to satisfy the requirements described in the previous subsections, for each rhythm, we now derive certain conditions on the set of inputs $I = \{ i_{\mathit {IP}}^{\mathrm {ext}}, i_{\mathit {EP}}^{\mathrm {ext}}, i_{\mathit {ER}}^{\mathrm {ext}}, i_{\mathit {IR}}^{\mathrm {ext}} \}$, which ensure that that rhythm will be selected. Some of these conditions are necessary, while together the collection is sufficient, although we cannot rule out that there may be different necessary and sufficient conditions elsewhere in parameter space. At a minimum, it is always necessary that the inputs actually elicit oscillations, both at the interneuron and the motoneuron levels. For convenience in what follows, define $T_{\mathrm {active}}^{j}(I)$ as the length of time for which population *j* is active for a given set of input parameters *I* as above.

Recall that we have defined a slow phase plane structure in which activation occurs by gaining access to the curve of jump up knees with *ER* active (as discussed in the previous subsection). For simplicity, we henceforth refer to $s_{\mathrm {dynamic}}$ as *s*. We define the interval $I_{s} = [s_{\mathit {ER}}^{\min}(I), s_{\mathrm {SN}}]$. $s_{\mathrm {SN}}$ is defined as the value of *s* at which the saddle-node bifurcation of fast subsystem critical points occurs with *ER* active (Figs. 8 and 9), and $s_{\mathit {ER}}^{\min}(I)$ is simply the minimum value to which *s* decays while *EP* is still active. The dependence of $s_{\mathit {ER}}^{\min}$ on input arises because the set *I* determines how long *EP* and *ER* are active and hence how far *s* decays from $s_{\max}$. The interval $I_{s}$ is illustrated for a particular input set *I* in Fig. 8.

When there is a switch between *EP* active and *ER* active, *s* jumps to $s_{\max}$. (This occurs instantaneously in the singular limit, but in our simulations, such as Figs. 8 and 9, the switch occurs at some $s^{*}< s_{\max}$. The value of $s^{*}$ can easily be approximated as $s^{*} \approx s_{\max}e^{-\beta t}$ where, using the differential equation for *s* in (1), *t* satisfies $s_{\max}e^{-\beta t} = (s^{\min}(I)-s_{\max})e^{-(\alpha +\beta)t} + s_{\max}$ given the minimal value of $s_{\mathrm {dynamic}}$ is $s^{\min}(I)$. This equality illustrates how $t \to0$ and hence $s^{*} \to s_{\max}$ as $\alpha\to\infty$, corresponding to a complete separation of time scales.) We assume that $h_{\mathit {EP}}^{\mathrm {JD}}(s_{\max})=h_{\mathit {ER}}^{\mathrm {JD}}(s_{\max})$ and denote this *h*-value by $h_{\max}$. This assumption is based on the numerical observation that the curves of right knees corresponding to *EP* active or *ER* active are quite close, which relates to the reversal of synaptic excitation at large voltages, and appear to converge at *s* near $s_{\max}$. We define a second interval $I_{h} = [ h_{\min}(I), h_{\max}]$, where $h_{\min}(I)$ is the value of $h_{\mathit {KE}}$ along the *ER* curve of jump down knees at $s=s_{\mathit {ER}}^{\min}(I)$. This interval specifies the full set of $h_{\mathit {KE}}$ values from which a jump down will yield a crossing of the curve of knees. The interval $I_{h}$ is illustrated for a particular input set *I* in Fig. 9.

##### Pocket Rhythm

**Fig. 10:**
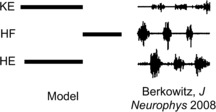
Pocket rhythm: duration and timing of MN activations in simulations (*left*) and experimental recordings from MNs (*right*). Recall that *HF* activates with *ER* and *HE* with *EP*

Recall the form of the pocket rhythm, illustrated in Figs. 8 and 10. Since *HE* is active longer than *HF* in this rhythm, we take $i_{\mathit {ER}}^{\mathrm {ext}}< i_{\mathit {EP}}^{\mathrm {ext}}$, which leads to $T_{\mathrm {active}}^{\mathit {ER}}(I)< T_{\mathrm {active}}^{\mathit {EP}}(I)$. In a successful pocket rhythm, *KE* activation can occur at any value of $s_{\mathrm {dynamic}}=s_{\mathit {ER}} \in I_{s}$. The closer to $s_{\mathit {ER}}^{\min}(I)$ that activation occurs, the less the overlap of *KE* and *HF* activations. With the above constraints and definitions, the pocket rhythm will exist for any set of inputs for which $I_{s}$ is mapped to $\operatorname {int}(I_{s})$ under the slow flow pieced together by appropriate selection of (3) and (4). This mapping to the $\operatorname {int}(I_{s})$ helps ensure that requirement (ii) in the previous section is met, as we will show below.

By continuity, it is sufficient for the existence of a stable pocket rhythm to find conditions on *I* under which the endpoints $s_{\mathrm {SN}}$ and $s_{\mathit {ER}}^{\min}(I)$ are mapped into the interior of $I_{s}$. We use slow phase plane arguments to do so. Fix input set *I*. Note that there is an ordering of the trajectories starting from the relevant part of the cyan curve of jump up knees corresponding to *ER* active (Fig. 8), given by $\mathit {LK}_{I_{s}} := \{ (h_{\mathit {KE}},s) : s \in I_{s}, h_{\mathit {KE}}=h^{\mathrm {JU}}_{\mathit {ER}}(s) \}$. That is, suppose $(h_{1},s_{1}), (h_{2},s_{2}) \in \mathit {LK}_{I_{s}}$ with $h_{1} > h_{2}$ and hence $s_{1}>s_{2}$. Flow $(h_{1},s_{1})$ forward under (3), obtaining a trajectory $(h_{1}(t),s_{1}(t))$, until $s_{1}(t)=s_{2}$. Similarly, denote the forward flow from $(h_{2},s_{2})$ as $(h_{2}(t),s_{2}(t))$. If $h_{1}(t)>h_{2}$ ($h_{1}(t)< h_{2}$), then $h_{1}(t+\tau)>h_{2}(\tau)$ ($h_{1}(t+\tau)< h_{2}(\tau)$) for all *τ* until $s_{1}(t+\tau)=s_{2}(\tau )=s_{\mathit {ER}}^{\min}(I)$ and the *ER* active phase ends. Moreover, by continuity, all points on $\mathit {LK}_{I_{s}}$ are ordered in this sense.

**Fig. 11:**
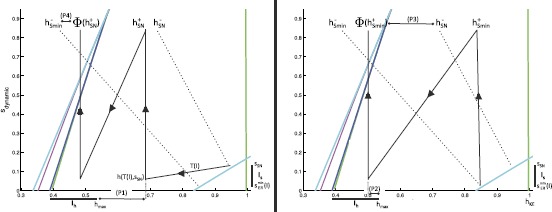
Useful trajectories for deriving sufficient conditions for a stable pocket rhythm. *Solid black lines* are flows forward from a known point. *Dotted black lines* represent backward flows. *Left*: the conditions that arise when a flow is initiated from $s_{\mathrm {SN}}$. *Right*: the conditions that arise when a flow is initiated from $s_{\mathit {ER}}^{\min}(I)$

Thus, the trajectory from $\mathit {LK}_{I_{s}}$ that attains the minimal $h(s)$ value when $s=s_{\mathit {ER}}^{\min}(I)$ when evolved forward in time is either the one starting from $s=s_{\mathrm {SN}}$ (corresponding to < in the statements above) or that from $s=s_{\mathit {ER}}^{\min}(I)$ (corresponding to >). It turns out that the more interesting case, for which our argument yields one additional sufficient condition, occurs when the minimal *h* corresponds to the initial condition $s=s_{\mathrm {SN}}$, with the initial value of *h* given by $h_{\mathrm {SN}}:=h^{\mathrm {JU}}_{\mathit {ER}}(s_{\mathrm {SN}})$, so without loss of generality we henceforth assume that this orientation holds (Fig. 11).

Now, let $T(I)=(1/\beta)\ln(s_{\mathrm {SN}}/s_{\mathit {ER}}^{\min}(I))$ denote the time for *s* to decay from $s_{\mathrm {SN}}$ to $s_{\mathit {ER}}^{\min}(I)$. Suppose we choose an initial condition such that *KE* activation occurs at $s=s_{\mathrm {SN}}$ during the *ER* active phase. We introduce the notation $h(a;b,c)$ to denote the $h_{\mathit {KE}}$ value at time *a* for a trajectory that started at time 0 with initial condition $(h_{\mathit {KE}},s)=(b,c)$. The first sufficient condition that we include is that the resulting *KE* trajectory does not cross a curve of jump down knees when *EP* takes over from *ER*:

$$\mbox{(P1)}\quad h_{\mathrm {SN}}^{+} := h\bigl(T(I);h_{\mathrm {SN}},s_{\mathrm {SN}} \bigr) > h_{\max}. $$

Condition (P1) ensures that the *KE* active phase overlaps with the active phase of *EP* and hence *HE*, as desired; in other words, $T_{\mathrm {active}}^{\mathit {KE}}(I)>T(I)$ (labeled in Fig. 11, left).

Next, we impose a condition to ensure that *KE* activation ends when *EP* activation does. This condition forces the *KE* trajectory with largest *h* value to lie in $I_{h}$ at the end of the *EP* active phase. This trajectory has initial condition $(h_{\mathit {ER}}^{\min}(I),s_{\max})$ at the start of the *EP* active phase, where $h_{\mathit {ER}}^{\min}(I):= h^{\mathrm {JU}}_{\mathit {ER}}(s_{\mathit {ER}}^{\min}(I))$, and evolves under (3) with *EP* active for time $T_{\mathrm {active}}^{\mathit {EP}}(I)$ (to $\{ s=s_{\mathit {EP}}^{\min}(I) \}$). The condition (Fig. 11, right) is

$$\mbox{(P2)}\quad h\bigl(T_{\mathrm {active}}^{\mathit {EP}}(I);h_{\mathit {ER}}^{\min}(I),s_{\max} \bigr) < h_{\max}. $$

Next, we obtain two conditions that are sufficient to ensure that the flow of $\mathit {LK}_{I_{s}}$ yields trajectories that return to $\operatorname {int}(\mathit {LK}_{I_{s}})$ and that do so while *ER* is active, but not newly active (to ensure requirement (ii) in the previous section). To state these conditions, we need to make use of the backwards flow of the endpoints $(h_{\mathrm {SN}},s_{\mathrm {SN}})$ and $(h^{\mathrm {JU}}_{\mathit {ER}}(s_{\mathit {ER}}^{\min}(I)),s_{\mathit {ER}}^{\min}(I))$ back to the line $\{ s = s_{\max} \}$ under system (3) with *ER* active. Denote the *h*-coordinates of these intersections by $h_{\mathrm {SN}}^{-}$ and $h_{s_{\min}}^{-}$, respectively, with $h_{s_{\min}}^{-} < h_{\mathrm {SN}}^{-}$ by continuity. Recall that the forward trajectory from the endpoint $(h_{\mathrm {SN}},s_{\mathrm {SN}})$ has $h = h_{\mathrm {SN}}^{+} := h(T(I);h_{\mathrm {SN}},s_{\mathrm {SN}})$ when *EP* becomes active (see Condition (P1) and Fig. 11, left). With these definitions, the final sufficient conditions, which guarantee that the next *KE* activation occurs from $\operatorname {int}(\mathit {LK}_{I_{s}})$, read

$$\begin{aligned} &\mbox{(P3)}\quad h\bigl(T_{\mathrm {active}}^{\mathit {EP}}(I);h_{\mathit {ER}}^{\min}(I),s_{\max} \bigr) < h_{\mathrm {SN}}^{-}, \\ &\mbox{(P4)}\quad h\bigl(T_{\mathrm {active}}^{\mathit {EP}}(I);h_{\mathrm {SN}}^{+},s_{\max} \bigr) > h_{s_{\min}}^{-}. \end{aligned} $$

(P1)–(P4) are conditions on relative orderings of points in the slow phase space that may result under certain choices of *I*. To appreciate that when *I* is chosen to satisfy Conditions (P1)–(P4), together with the earlier condition that $T_{\mathrm {active}}^{\mathit {ER}}(I) < T_{\mathrm {active}}^{\mathit {EP}}(I)$, it follows that $\mathit {LK}_{I_{s}}$ is mapped into its own interior under the flow and there exists a stable periodic pocket rhythm, note that the time of evolution from $s=s_{\max}$ down to $s=s_{\mathit {ER}}^{\min}(I)$ under (3) with *EP* active is exactly time $T_{\mathrm {active}}^{\mathit {EP}}(I)$. Conditions (P3)–(P4) ensure that all trajectories emanating from $\mathit {LK}_{I_{s}}$ end up with $h \in(h_{\min}^{-},h_{\mathrm {SN}}^{-})$ when *ER* first activates. From the time of *ER* activation, these trajectories all evolve under (3) from $s=s_{\max}$, and Conditions (P3)–(P4) imply that they reach $\operatorname {int}(\mathit {LK}_{I_{s}})$. In particular, they arrive with $s>s_{\mathit {ER}}^{\min}(I)$ and hence they do so after times that are less than $T_{\mathrm {active}}^{\mathit {ER}}(I)$, before the end of the *ER* active phase, as desired. Furthermore, Condition (P3) allows us to clarify what we mean by “sufficiently late” in requirement (ii) from the previous section. That is, the time *KE* spends in the silent phase is minimized when it activates from $(h_{\mathrm {SN}}, s_{\mathrm {SN}})$, or equivalently when it enters the silent phase at $h=h_{\mathrm {SN}}^{-}$. We can use Eq. (3) to calculate the minimal time spent in the silent phase: $t^{*} = \frac{-1}{\beta} \ln( \frac{s_{\mathrm {SN}}}{s_{\max}})$. (P3) guarantees that *ER* is active for at least time $t^{*}$ before *KE* activates.

In summary, we conclude that for a choice of synaptic weights such that our earlier assumptions on the structure of phase space are satisfied, for any choice of *I* such that Conditions (P1)–(P4) hold, there exists an open set of initial conditions supporting a stable, periodic pocket rhythm. Choices of weights that shrink $s_{\mathrm {SN}}$ toward $s_{\mathit {ER}}^{\min}(I)$, narrowing $I_{s}$, yield less overlap between the phases when *KE* and *HF* are active at the end of the *ER* active phase, and hence more experimentally realistic solutions. This change can be achieved, for example, by weakening the excitation from *ER* to *KE* relative to the inhibition from *IP* to *KE*; however, making this excitation too weak will prevent *KE* activation entirely and destroy the rhythm.

##### Rostral Rhythm

Next, recall the form of the rostral rhythm, illustrated in Fig. 9. Since *HF* is active longer than *HE* in this rhythm, we take $i_{\mathit {EP}}^{\mathrm {ext}}< i_{\mathit {ER}}^{\mathrm {ext}}$, which leads to $T_{\mathrm {active}}^{\mathit {EP}}(I)< T_{\mathrm {active}}^{\mathit {ER}}(I)$. In the rostral rhythms that we seek, we assume that *KE* activation occurs with $s_{\mathrm {dynamic}}=s_{\mathrm {SN}}$ with *ER* (and thus *HF*) active, in order to achieve the delay with respect to *HF* activation in a robust way, keeping the same synaptic weights as in the pocket case. We also require that *KE* activation ends at the same time as *ER* activation. We now use slow phase plane arguments to derive sufficient conditions for the existence of a stable rostral rhythm that meets these constraints.

**Fig. 12:**
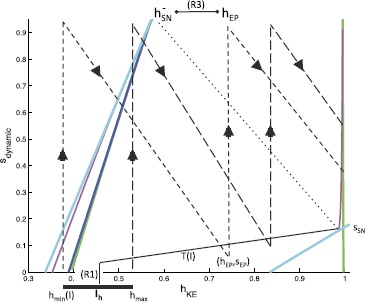
Useful trajectories for deriving sufficient conditions for a stable rostral rhythm. *The solid black line* denotes the flow forward from $(h_{\mathrm {SN}},s_{\mathrm {SN}})$. *Dashed black lines* indicate flows forward from two points $(h_{\max}, s_{\mathrm {SN}})$ and $(h_{\min}(I),s_{\mathrm {SN}})$. *The dotted black line* represents a backward flow

The trajectory for the desired rhythm should reach the curve of jump up knees with $s=s_{\mathrm {SN}}$ and *ER* active and flow from there to the interval $I_{h}$. Using our previous definitions of $T(I)$ and $h_{\mathrm {SN}}$, a sufficient condition to achieve this requirement is simply (Fig. 12):

$$\mbox{(R1)}\quad h\bigl(T(I);h_{\mathrm {SN}},s_{\mathrm {SN}}\bigr) \in I_{h}. $$

Next, it suffices to impose conditions under which the flow maps the interval $I_{h}$ back to the curve of jump up knees where it intersects $\{ s=s_{\mathrm {SN}} \}$ at some time after *ER* has already activated but while *ER* is still active. To derive these, it suffices to consider the trajectories generated by the forward flow from the endpoints of $I_{h}$, namely $(h_{\min}(I),s_{\mathit {ER}}^{\min}(I))$ and $(h_{\max},s_{\mathit {ER}}^{\min}(I))$. There are two aspects to this mapping requirement. One is that all trajectories have time to reach $\{ s=s_{\mathrm {SN}} \}$ from $\{ s = s_{\max} \} $ (Fig. 12), a condition for which can be written in two equivalent forms using the notation we have introduced:

$$\mbox{(R2)}\quad s_{\mathrm {SN}} > s_{\mathit {ER}}^{\min}(I) \quad \Leftrightarrow\quad T_{\mathrm {active}}^{\mathit {ER}}(I) > (1/\beta) \ln(s_{\max}/s_{\mathrm {SN}}). $$

The other aspect is that even the trajectory with minimal *h* value, which originates from $(h_{\min}(I),s_{\mathit {ER}}^{\min}(I))$ just before *EP* activates, must be able to reach $(h_{\mathrm {SN}},s_{\mathrm {SN}})$ while *ER* is active. This trajectory flows forward from $(h_{\min}(I),s_{\max})$ under (3) with *EP* active, say to $(h_{\mathit {EP}},s_{\mathit {EP}})$, and then continues forward under (3) with *ER* active from $(h_{\mathit {EP}},s_{\max})$ (Fig. 12). Our additional sufficient condition is therefore

$$\mbox{(R3)} \quad h_{\mathit {EP}} > h_{\mathrm {SN}}^{-}, $$

where $h_{\mathrm {SN}}^{-}$ is derived from the backwards flow of (3) with *ER* active as in the previous subsection.

Conditions (R1)–(R3), together with the earlier condition that $T_{\mathrm {active}}^{\mathit {EP}}(I) < T_{\mathrm {active}}^{\mathit {ER}}(I)$, are sufficient for all initial conditions within $I_{h}$ to pass through $(h_{\mathrm {SN}},s_{\mathrm {SN}})$, in the singular limit, albeit at different times, and reach the interior of $I_{h}$ with *ER* active, which guarantees a stable rostral rhythm. We observe that our strong structural requirement that *KE* activation occurs at a saddle-node bifurcation of fast subsystem equilibria, which ensures a robust delay of *KE* activation relative to *ER* (and hence *HF*) activation as seen in the rostral rhythm, makes our remaining sufficient conditions for the existence of a stable rostral rhythm milder than those we invoked to ensure the existence of a stable pocket rhythm.

##### Key Differentiator Between Rhythms

The work in this section supplies a variety of conditions on the relative positions of various trajectories such that when a set of inputs allows an appropriate collection of conditions to be satisfied, a pocket or rostral rhythm results. From this analysis and our numerical simulations, we can extract a key factor that distinguishes whether a rhythm generated by an input set is likely to be a pocket rhythm or a rostral rhythm. Given an initial condition on $\mathit {LK}_{I_{s}}$ with *ER* active,

- inputs that lead to $h_{\mathit {KE}}>h_{\max}$ at the termination of *ER* activity push the solution toward pocket;
- inputs that lead to $h_{\mathit {KE}}< h_{\max}$ at the termination of *ER* activity push the solution toward rostral.

**Fig. 13:**
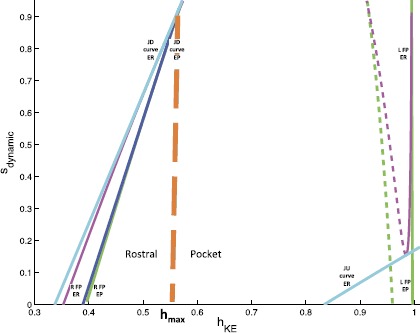
Key differentiator. The location of a trajectory at the end of the *ER* active phase, relative to $h_{\max}$, ends up being the key separator in the slow phase plane between inputs that elicit rostral and those that elicit pocket

In other words, roughly speaking, the rhythm is selected based on whether or not the *KE* trajectory has access to a curve of jump down knees from which to enter the silent phase at the switch from *ER* activity to *EP* activity (Fig. 13). Of course, this access depends on the time remaining with *ER* active after *KE* activates, which in turn depends on all relationships presented in the previous two subsections. Nonetheless, a numerical exploration of this timing issue can give a quick, rough idea of which solutions will be favored for a given input set, an option that would not have been obvious without our analysis. Further, this analysis provides a framework in which features can be examined thoroughly, which we harness in the next section.

### Modeling Additional Experimental Results

#### Experiments and Simulations with Input Switching

We can test the experimental relevance of our model by trying to simulate some additional experiments that have been performed involving the rostral and pocket rhythms. Furthermore, now that we understand the dynamic mechanisms underlying each rhythm and the rhythm selection process, we can understand the outcomes of simulations in these scenarios.

**Fig. 14:**
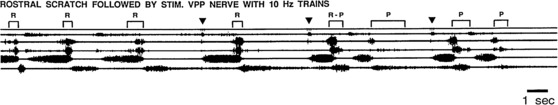
Currie and Stein 1988 experiments. Converting a rostral rhythm to a pocket rhythm. *Bottom three traces* show MN activity corresponding to *KE*, *HF*, and *HE*, respectively. Initial bouts of activity represent a rostral rhythm with large delay of *KE* activation relative to *HF*. Transient pulse stimulation of the VPP nerve (*inverted triangles*) eventually switches the network into a pocket rhythm. Figure source: [31]

In their 1988 work seeking to further typify scratch and swim behavior, Currie and Stein [31] explored the presentation of rhythm-specific stimulation during ongoing scratch activity. For example, while the turtle was exhibiting the rostral scratch pattern (following stimulation in the rostral body region), stimulation was provided in the pocket body region, which could eventually lead to a period of blended rhythm, followed by the pocket scratch (Figs. 1 and 14).

**Fig. 15:**
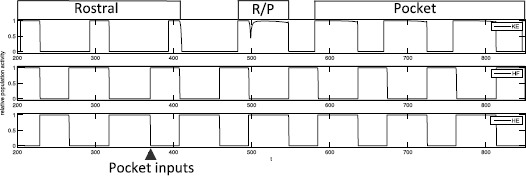
Simulation of Currie and Stein 1988 experiments. A switch from rostral inputs to pocket inputs, at the time indicated by *the arrow*, causes the model behavior to transition from rostral to blended output to pocket. Standard weights were used, with similar results obtained for SCE weights (not shown). Inputs: $I_{\mathrm {rostral}}= \{i_{\mathit {IP}}=0.19, i_{\mathit {EP}}=0.17, i_{\mathit {ER}}=0.19, i_{\mathit {IR}}=0.17\}$, $I_{\mathrm {pocket}}= \{i_{\mathit {IP}}=0.17, i_{\mathit {EP}}=0.19, i_{\mathit {ER}}=0.17, i_{\mathit {IR}}=0.19\}$

To qualitatively reproduce this experiment, we consider the result of an instantaneous switch of inputs. That is, a rostral input set, $I_{\mathrm {rostral}}$, is given to the system. After several periods, at the end of a phase of *HE* activity (as in the experiment), the inputs are switched to a pocket input set, $I_{\mathrm {pocket}}$. With both the Standard and the Strong Cross-Excitation synaptic weights, this change in inputs leads to a similar transition to pocket as seen in the experiment (Fig. 15). Our phase plane analysis makes it easy to understand the switch in dynamics. Once pocket inputs are applied, *KE* still reaches the SN bifurcation and activates while *EP* and *HF* are active, as in rostral. But the pocket inputs shorten $T_{\mathrm {active}}^{\mathit {ER}}(I)$, allowing *EP* and hence *HE* to take over before $h_{\mathit {KE}}$ decays down to $h_{\max}$. Thus *KE* remains active when $\mathit {EP}/\mathit {HE}$ activates, yielding a cycle that blends features of rostral and pocket followed by rapid convergence to a pocket rhythm.

**Fig. 16:**
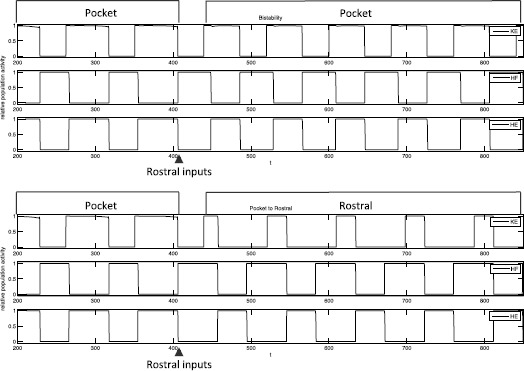
Pocket to rostral simulations. Applying rostral inputs during a pocket rhythm may or may not induce a switch to rostral. A pocket rhythm was induced using $I_{\mathrm {pocket}}=\{i_{\mathit {IP}}=0.17, i_{\mathit {EP}}=0.19, i_{\mathit {ER}}=0.17, i_{\mathit {IR}}=0.19\}$. Inputs were switched at the time indicated by *the arrows* to one of two different input sets, each of which evoked rostral from rest. *Top*: $I_{\mathrm {rostral}}^{1} = \{i_{\mathit {IP}}=0.19, i_{\mathit {EP}}=0.18, i_{\mathit {ER}}=0.19, i_{\mathit {IR}}=0.18\}$ maintains the pocket rhythm, and hence uncovers bistability in the system. *Bottom*: $I_{\mathrm {rostral}}^{2}=\{i_{\mathit {IP}}=0.19, i_{\mathit {EP}}=0.17, i_{\mathit {ER}}=0.19, i_{\mathit {IR}}=0.17\}$ leads to switching behavior as seen in experiments [31]

We also consider the reverse scenario of applying rostral inputs during an ongoing pocket rhythm. Interestingly, simulations of this manipulation yield different results depending on whether we use our Standard or SCE synaptic weights. In the Standard set up, interrupting pocket at the end of an *HE* cycle with two different input sets, each of which yields a rostral rhythm when applied to the model in a rest state, induces two qualitatively different behaviors. In one case, even with the rostral inputs, a rhythm that can be classified as pocket persists, although *HF* is active slightly longer than *HE*, unlike the prototypical pocket rhythm (Fig. 16, top). In the other case, the rostral inputs cause a switch to the rostral rhythm (Fig. 16, bottom).

In the case where pocket persists, we conclude that the rostral inputs that are applied render the system bistable. These inputs are closer to $I_{\mathrm {pocket}}$ than are other rostral inputs that do not reveal bistability. In particular, the stronger inputs to *IR* and *EP* in the former case cause an earlier switch from *HF* to *HE*, allowing pocket dynamics to be maintained. In the SCE set up, we do not observe bistability numerically across a wide range of inputs and synaptic weights that we have explored.

#### Explanation of Bistability (and Lack Thereof)

The selection between the two cases illustrated in Fig. 16 essentially comes down to a race between *EP* (corresponding to *HE*) and *KE*: from the activation of $\mathit {ER}/\mathit {HF}$, does *EP* reach the jump up knee before $h_{\mathit {KE}}$ is able to decay to reach $h_{\max}$? If *EP* does activate first, then the rhythm remains in pocket. If *KE* reaches $h_{\max}$ first, then a switch to rostral can occur. The data used to generate Fig. 16 indicates that a decrease in $i_{\mathit {EP}}$ promotes this switch. This idea can be investigated more closely through a series of numerical calculations of these quantities, with a few approximations motivated by the framework that the slow phase plane analysis provides.

In the SCE regime, we have not observed bistability: introduction of rostral inputs during ongoing pocket switches the rhythm to rostral. Heuristically, we can see why SCE would tend to suppress bistability, based on the SCE synaptic weights (Fig. 5). For a pocket rhythm to persist despite rostral inputs, the *ER* active phase must remain sufficiently short that *EP* can activate before $h_{\mathit {KE}}$ drops to $h_{\max}$ (Fig. 13). Because transitions in our networks occur by escape, this requirement means that *EP* or *IR* must be able to activate before the *ER* stays active too long. In SCE, however, the weights of synaptic inhibition from *ER* to *IR* and excitation from *ER* to *IP* are strong, relative to the S case. These synaptic connections are exactly the ones that would suppress the activation of *EP* and *IR* and thus prolong the *ER* active phase, causing *KE* to jump down with *ER* and inducing a switch from pocket to rostral.

#### Predictions

The observation that some weight and input parameter sets yield bistability and others do not may be useful for making predictions. That is, if bistability is observed experimentally, then we can conservatively state that it should rule out certain parameter combinations within the underlying rhythm generating circuit, if indeed that circuit is qualitatively represented by our model. For example, although our simulations were not exhaustive, together with the heuristic arguments we have provided they suggest that an observation of bistability of pocket and rostral rhythms in response rostral inputs would represent evidence against SCE weights, in which both the excitatory and the inhibitory interneurons projecting to *HF* are more strongly recruited by rostral stimulation than are the corresponding *HE*-projecting interneurons.

More generally, we can also observe that if a single circuit generates both pocket and rostral rhythms, then one rhythm may be more resistant to input-induced switching than the other, as we have seen by introducing rostral input during an ongoing pocket rhythm. This is an important observation: Suppose that two separate modules generated pocket and rostral rhythms. In that case, introducing a rostral input during ongoing pocket would necessarily recruit the rostral module, likely perturbing the pocket rhythm in some way that is more significant than seen in our simulations. Hence, bistability may be used to help distinguish between these possible rhythm generation frameworks (see also [5]).

**Fig. 17:**
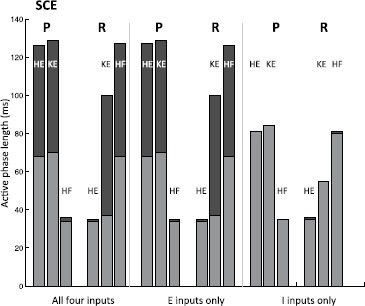
Effect of input scaling on phase durations in SCE regime. *The black bars* represent the durations of the active phases of *HE*, *KE*, and *HF* when the indicated inputs are uniformly decreased by multiplication by a scaling factor less than one, just large enough to maintain each rhythm. *The gray bars* represent the durations of the active phases of *HE*, *KE*, and *HF* when the scaling factor is greater than one, near the upper bound for maintaining each rhythm

Additionally, we can consider the effect of scaling inputs to the interneurons. We consider what happens in the SCE regime when all four inputs are scaled by the same factor, only the E inputs (to *EP*, *ER*) are scaled by the same factor, or only the I inputs (to *IP*, *IR*) are scaled by the same factor (Fig. 17). In the first case (Fig. 17, left), we see that increasing inputs (black to gray) leads to a decrease in active phase length for both *KE* and the dominant IN population (namely *HE* in pocket and *HF* in rostral) with almost no change in phase duration for the other population. This result, which is consistent with the stipulation that phase transitions occur by escape and also with past work exploring asymmetries in persistent sodium half-center oscillator models [18, 26], represents a testable prediction. Next, we find that scaling only the inputs to the excitatory INs leads to almost the same changes in active phase durations as occur when all inputs are scaled (Fig. 17, left versus middle), while there is virtually no change in active phase length across different scalings of the inputs to the inhibitory INs (Fig. 17, right). These results indicate that the escape of the excitatory INs from the silent phase largely controls rhythm frequencies. In fact, we find that the external input to the inhibitory interneurons can be removed and the synchrony patterns of the rhythms (but not the delay in rostral) can be maintained (data not shown), because the excitatory INs still recruit the inhibitory populations to become active. These predictions are more difficult to test, given that these populations of interneurons have not yet been identified, but remain for future experimental consideration.

**Fig. 18:**
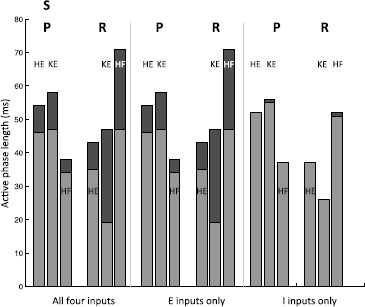
Effect of input scaling on phase durations in S regime. *The black bars* represent the durations of the active phases of *HE*, *KE*, and *HF* when the indicated inputs are uniformly decreased by multiplication by a scaling factor less than one, just large enough to maintain each rhythm. *The gray bars* represent the durations of the active phases of *HE*, *KE*, and *HF* when the scaling factor is greater than one, near the upper bound for maintaining each rhythm

We repeat this experiment with the S regime (Fig. 18) and find generally very similar results. However, it is worth noting that, in the S regime, the changes in active phase durations across similar scaling is much less than in the SCE regime. Additionally, there is a much greater change in active phase durations in rostral than in pocket. These differences, in addition to the bistability observed, may serve to differentiate the S regime from the SCE regime in practice.

## Discussion

It has been postulated that turtle scratching and swimming arise when “behavioral modules” interact and combine to control “muscle synergies” producing appropriately coordinated motor outputs [32], but there is a large gap between such an abstract statement and concrete hypotheses about the neuronal networks involved. While a specific wiring diagram for a single circuit that could parsimoniously drive both pocket and rostral scratching has been proposed [4], it is well known that connectivity diagrams alone do not uniquely map to output patterns [17]. We have performed a computational and mathematical study to investigate whether the proposed unified CPG network, which features only hip-related populations of interneurons, could indeed be responsible for the generation of two different turtle scratch rhythms with distinct knee-hip synchrony patterns. Importantly, these patterns are selected by changing external inputs to the interneurons, with the same synaptic weights between interneurons, and from interneurons to motoneurons, preserved for both. Through the use of slow phase plane arguments, we were able to explain how particular phase space and bifurcation structures underlie the generation of the rhythms and to derive sufficient conditions on these structures that guarantee the existence of stable rhythms. This analysis was possible due to time scale decomposition and certain model reductions, despite the relative high-dimensionality of the model system; because our conditions are stated in terms of dynamic structures, they apply beyond the particular model features, such as a slowly inactivating persistent sodium current, used in our simulations. Even with model reductions, the synaptic variables evolving during each stage of each rhythm were hybrid variables, representing combined effects of excitatory and inhibitory inputs, which was one unusual aspect of our analysis.

Past research has focused on several different aspects that arise in multi-functionality, including the general organizing principles governing CPGs [16, 33], and the notion that an organism exhibits different motor patterns by selecting different CPGs [34], which may be collections of burst-capable unit CPGs that each control a set of synergistic muscles [11]. Similarly, recent experimental work in mice [13] found that the hindlimb locomotor network is composed of intrinsically rhythmic modules that each drive a pool of motoneurons. Consistent with the unit CPG framework, the model that we consider includes separate hip extensor and hip flexor interneuron pairs (*EP* and *IP*, *ER* and *IR*); although each individual population is tonically active in the absence of inputs, each pair can generate bursts through a mechanism of escape from reciprocal inhibition, consistent with previous related work [5]. Our interneuron network includes fixed interconnections and projections both to antagonist hip interneurons and to hip motoneurons and is able to generate multiple rhythms under changes in inputs that alter the relative durations of the unit CPGs, without changing network connections. In contrast to the unit CPG idea, however, the hip interneurons also control knee extensor motoneurons in the model. Despite the multi-tasking demanded of the unit CPGs, we find that the network can generate multiple motor patterns, selected by tuning the relative strengths of their tonic inputs. That networks of unit CPGs can be influenced to demonstrate different activity patterns is not surprising, given the wide variety of activity patterns that can be elicited from a single neuron [35, 36], but the idea that CPGs for one unit can also be harnessed to control the timing of another joint is relatively novel. Although this idea makes sense in terms of efficient use of neuronal resources, evolutionary principles, and the observation that individual interneurons are active during multiple forms of activity [1], it remains to be determined whether this framework offers enough robustness for functional rhythm generation.

A distinctive feature of one of the rhythms considered, the rostral scratch, is a delay in the onset of *KE* motoneuron activity relative to *HF* onset. While synchronization ([27, 37]) and near-synchronization [38] in networks of planar neuron models with strong synaptic coupling has been well studied, the delay we consider appears to be novel. This delay significantly restricts the choices of synaptic weights to *KE* for which both rhythms can be elicited. The resulting phase plane structure leads us to observe that, given that the sufficient conditions on synaptic weights hold, the rhythm selected by a particular input set is largely determined by the position of the slow variable coordinate of a particular trajectory segment relative to a key value $h_{\max}$ at the termination of *ER* activity (Fig. 13).

Unfortunately, from an experimental point of view, the specific rhythm generation conditions in our model are not accessible for many reasons, starting with the fact that the interneuron populations in the CPG have not been identified. However, our analysis yields the observation that in the framework we have considered, the *KE* motoneuron must activate slightly before the *HE* during the pocket rhythm, and this is exactly what is observed experimentally [30], which offers some validation for our approach. Furthermore, simulation of the model can help guide future experiments. In particular, the model network can exhibit bistability to rostral scratch inputs for some of the parameter values considered, which seems unlikely to arise with separate pocket and rostral generation modules (see also [5]). Thus, future experiments to explore this form of bistability could be useful. The slow phase plane approach that we have presented provides a framework that can be used to make predictions about specific experiments and to explain the mechanisms underlying observed outcomes. Our simulations also predict that changes in inputs to the CPG that are not strong enough to destroy an ongoing rhythm will alter the active phase durations of the hip MN that is dominant in that rhythm and of the knee extensor MN while leaving the other hip MN activity period almost entirely unchanged, and that these changes are controlled by the excitatory INs in the CPG. These outcomes likely result from the underlying assumption that activity transitions in our model occur through escape [25, 26], based on past experimentally constrained work modeling turtle motor CPGs [5], and alternative transition mechanisms should be considered if these predictions are falsified in future experiments.

During rostral scratching, hip extensor deletions can occur [9, 39]. In these deletions, hip extensor is silent while knee extensor behavior is entirely preserved (synchrony with hip flexor after a delay, periods of full activity and full quiescence); hip flexor fails to shut down fully during its quiescent period, as during normal rostral. This lack of quiescence presumably results from the absence of inhibition from hip extensor related motor pools. These deletions occur unpredictably in some preparations, although the frequency can be increased through particular experimental techniques [9]. Due to a combination of the proposed architecture and the use of deterministic differential equations to describe population behavior, it is not possible to reproduce this behavior fully in our model. The only way to shut down hip extensor behavior in both the standard and the strong cross-excitation architectures, by only changing inputs and without changing synaptic weights from interneuron motor pools to the hip extensor (which would be a trivial but non-biological solution), is by decreasing input to *IR* and *EP* until oscillations are lost (Fig. 2). While this does lead to tonic activity in hip flexor as desired, it also leads to tonic activity in knee extensor. One possible way to resolve this issue is to suppose that an additional source of inputs, not included in our model, provides enough inhibition to shut down knee extensor motoneurons while the *ER* input is low. A need to invoke additional inputs to explain deletions suggests that hip-related motor pools may account for synchrony and relative timing of scratch rhythms but may not be sufficient to fully capture all motor behaviors observed. Although experiments suggest that inputs to knee extensor motoneurons are hip related, it may be that knee motor pools (as in the standard UPG approach to rhythm generation) are present in a secondary role and that interneurons related to knee flexor activity provide inhibition that contributes to the termination of knee extensor activity; past experiments on pocket and rostral scratch have not focused on knee flexor motoneuron recording [1–4], and hence we omit knee flexion in our model, but it could be included in future work. Alternatively, stochasticity may need to be taken into account to capture the full range of scratch rhythm phenomenology [40, 41]. Certainly, our model could be expanded to include additional neuron pools or stochastic mechanisms. Additional experimental work to constrain the mechanisms underlying deletions would be beneficial to help guide efforts in this direction.

It has been suggested that oscillations underlying turtle motor rhythms may be driven by concurrent excitation and inhibition, based on analysis of data showing that the estimated synaptic conductances for excitation and inhibition to turtle motoneurons oscillate in phase [12]. It is worth noting, however, that for the most part, neither the type (hip extensor, hip flexor, and so on) of motoneurons from which recordings were obtained nor the source of synaptic inputs was identified, so it is hard to know how to interpret these results. Past reviews [16] hypothesize that this may be an artifact of the experimental setup or a feature unique to motor pattern generation in turtles (as opposed to say mammals). These findings contrast with the traditional reciprocal model in which motoneurons receive synaptic excitation and inhibition in antiphase [9, 26], as imposed by the mutual inhibition between *EP* and *IP* and between *ER* and *IR* in our model network. Note that we chose this mutually inhibitory structure on experimental grounds: It has long been established that *HE* is active together with its excitatory motor pool of interneurons, *EP*; additionally, *HE* and *HF* activate in antiphase (Fig. 1) [2]. The simplest way to meet these benchmarks is for *EP* to be active with *IR* and *ER* with *IP*, as imposed by mutual inhibition. Nonetheless, it would be interesting to explore how stochastic effects might allow multi-functionality of a rhythm generating circuit despite less segregated excitatory and inhibitory inputs to its motoneurons, especially since rhythm generation in several other CPG circuits involves some mixture of reciprocal and concurrent excitation and inhibition (see references in [9]).

Another important future challenge will be to bring this work together with other previous modeling efforts [5] to develop a system capable of generating all four observed motor patterns, forward swim and rostral, pocket, and caudal scratch. While it is possible to produce rhythms like forward swim and caudal scratch with a hip dominated architecture as explored in this work, different synaptic weights (and, therefore, a different network) are necessary. It is an open problem to ascertain whether a single network could produce all four patterns. One possible approach to this problem would be the use of genetic algorithms to derive optimal CPG network structures [42, 43] or to determine the parameter values necessary to coordinate multiple CPGs to generate multiple rhythms [34]. It is not clear what would constitute a practically useful objective function for a genetic algorithm approach, however. Including more of the known details about the ionic currents in turtle motoneurons [23] would be another way to tie our modeling more closely to the biology of turtle motor rhythms in future works. Finally, it is worth considering the effect of within-leg proprioceptive sensory feedback, as is often considered with cats [8, 16, 44]. However, at present such data appears to be unavailable in the literature regarding turtles. Future work could include testing hypotheses about the effects of feedback in the present model, to yield predictions for future experimental testing.
